# Pastas: Open Source Software for the Analysis of Groundwater Time Series

**DOI:** 10.1111/gwat.12925

**Published:** 2019-08-24

**Authors:** Raoul A. Collenteur, Mark Bakker, Ruben Caljé, Stijn A. Klop, Frans Schaars

**Affiliations:** ^1^ Water Management Department, Faculty of Civil Engineering and Geosciences Delft University of Technology, Stevinweg 1 2628 CN Delft The Netherlands; ^2^ Artesia Water, Korte Weistraat 12 2871 BP Schoonhoven The Netherlands; ^3^ Witteveen and Bos, Blaak 16 3011 TA Rotterdam The Netherlands

## Abstract

Time series analysis is an increasingly popular method to analyze heads measured in an observation well. Common applications include the quantification of the effect of different stresses (rainfall, pumping, etc.), and the detection of trends and outliers. Pastas is a new and open source Python package for the analysis of hydrogeological time series. The objective of Pastas is twofold: to provide a scientific framework to develop and test new methods, and to provide a reliable ready‐to‐use software tool for groundwater practitioners. Transfer function noise modeling is applied using predefined response functions. For example, the head response to rainfall is simulated through the convolution of measured rainfall with a Gamma response function. Pastas models are created and analyzed through scripts, ensuring reproducibility and providing a transparent report of the entire modeling process. A Pastas model can be constructed in seven simple steps: import Pastas, read the time series, create a model, specify the stresses and the types of response functions, estimate the model parameters, visualize output, and analyze the results. These seven steps, including the corresponding Python code, are applied to investigate how rainfall and reference evaporation can explain measured heads in an observation well in Kingstown, Rhode Island, USA. The second example demonstrates the use of scripts to analyze a large number of observation wells in batch to estimate the extent of the drawdown caused by a well field in the Netherlands. Pastas is free and open source software available under the MIT‐license at http://github.com/pastas/pastas.

## Abstract 2

*Article impact statement*: Perform time series analysis of observed heads in a few lines of Python code with the open source software Pastas.

## Introduction

Over the past few decades, time series analysis has become an accepted and frequently applied methodology in the field of groundwater hydrology. A particularly popular subdiscipline of time series analysis is transfer function noise (TFN) modeling, which attempts to translate one or more input series to an output series using a statistical model. TFN models can be used, for example, to decompose observed head time series into the contributions of the different hydrological stresses that cause the head fluctuations. Applications of TFN models include the estimation of the effect of interventions in the groundwater system (such as groundwater pumping and changes in surface water levels), the detection of trends, the improvement of data quality by identifying outliers and other suspicious data values, and the forecasting of heads.

The first TFN models used in hydrogeology were autoregressive‐moving average models, which originate from econometrics (see Box and Jenkins 1970; Gehrels et al. 1994; Hipel and McLeod 1994). These models were extended with Kalman filters to deal with the irregular timesteps often found in time series of heads measured in an observation well (e.g., Berendrecht et al. 2004). Von Asmuth et al. (2002) introduced a new type of TFN models based on the principles of convolution and predefined impulse response functions. This type of model has been applied in a variety of studies including the decomposition of hydrological stresses (von Asmuth and Knotters 2004; von Asmuth et al. 2008; Shapoori et al. 2015b), the estimation of aquifer parameters (e.g., Obergfell et al. 2013; Shapoori et al. 2015a), the statistical interpolation of groundwater time series (Peterson et al. 2018), the analysis of nation‐wide groundwater monitoring networks (Zaadnoordijk et al. 2018), and the estimation of recharge (Obergfell et al. 2019).

The concepts of TFN modeling based on impulse response functions have been incorporated in the commercially available software Menyanthes (von Asmuth et al. 2012) and the open source software HydroSight (Peterson and Western 2014). Both programs are written in Matlab and are operated through a graphical user interface (GUI), while HydroSight also offers the possibility to run models through Matlab scripts. Autoregressive‐moving average models are readily available to hydrogeologists in popular open source programming languages such as R and Python (e.g., the Python package StatsModels, Seabold and Perktold 2010). Prior to this paper, no open source alternatives were available to perform TFN modeling based on impulse response functions in either R or Python. The new Python package Pastas is developed to fill this gap.

Pastas is an open source Python package to perform time series analysis of heads measured in an observation well. Python scripts are used to import data, construct models, optimize parameters, and postprocess the results. The use of scripts ensures reproducibility and provides a transparent report of the entire modeling process (Bakker 2014; Fienen and Bakker 2016; Hutton et al. 2016), in a similar way that FloPy (Bakker et al. 2016) can be used to construct a MODFLOW groundwater model. Pastas has an object‐oriented design that allows for the quick implementation of new modeling concepts. Combined with the ability to use scripts, this serves the main purpose of Pastas: to provide a scientific framework to improve existing methods or develop and test new methods, while at the same time provide a reliable ready‐to‐use software tool for groundwater practitioners in the field. A guiding principle during Pastas development is to give the user full control of the modeling process; sensible default values and options are selected for practitioners without restricting adventurous expert users.

The Pastas software and the workflow suggested in this paper fully comply with the four steps suggested by Hutton et al. (2016) to improve reproducibility in computational hydrology. First, the code is modularized into well‐documented functions and classes to make it readable and reusable. Second, the use of Python scripts and Jupyter Notebooks ensures well‐documented workflows that can be shared easily. Third, the source code is available on a Github repository which provides full version control of the software. And fourth, it is possible to refer to specific versions of the code by referring to a Pastas version and its related digital object identifier (DOI) on Zenodo (e.g., version 0.11.0, DOI: 10.5281/zenodo.3252035; Collenteur et al. 2019).

## Basics of TFN Modeling

TFN modeling tries to explain an observed time series (in this case observed heads) by one or more other observed time series. The basic model structure of a TFN model to simulate heads may be written as:

(1) $$h\left(t\right)=\sum_{m=1}^{M}h_{m}\left(t\right)+d+r\left(t\right)$$

where *h*(*t*) are the observed heads, *h*_*m*_(*t*) is the contribution of stress *m* to the head, *d* is the base elevation of the model, and *r*(*t*) are the residuals. Each model can have an arbitrary number of stresses (*M*) that contribute to the head; hydrological stresses include rainfall, evaporation, river levels, and groundwater extractions. The contribution of stress *m* to the head is computed through convolution:

(2) $$h_{m}\left(t\right)=\int_{-\infty }^{t}S_{m}\left(\tau \right)\theta_{m}\left(t-\tau \right)d\tau$$

where *S*_*m*_ is a time series of stress *m*, and *θ*_*m*_ is the impulse response function for stress *m*. A commonly used impulse response function is the scaled Gamma distribution (e.g., Besbes and De Marsily 1984):

(3) $$\theta \left(t\right)=A\frac{t^{n-1}}{a^{n}\;\Gamma \left(n\right)}e^{-t/a}\;t\geq 0$$

where *A* is the scaling factor, *a* and *n* are shape parameters, and *Γ* is the Gamma function. The scaled Gamma distribution is often used to simulate the response to areal recharge. Other impulse response functions have been suggested to simulate the effects of other stresses. For example, one of the response functions suggested for the response to pumping is the Hantush function (Hantush and Jacob 1955), which may be written in parametric form as:

(4) $$\theta \left(t\right)=-A\frac{t^{-1}}{2K_{0}\left(2\sqrt{b/a}\right)}e^{-t/a-b/t}\;t\geq 0$$

where *A* is a scaling factor, *a* and *b* are shape parameters, and K_0_ is the modified Bessel function of the second kind and order zero.

There are three types of response functions. The impulse response is the head response due to an instantaneous stress event of unit magnitude at time *t* = 0, for example an instantaneous precipitation event of unit amount. The head response due to a uniform stress is called the step response, for example, a well turns on and starts pumping with a constant discharge. The head response due to a uniform stress for 1 day is called the one‐day block response and can be obtained through superposition of two step responses.

The step response Θ(*t*) due to a constant and unit stress starting at *t* = 0 may be obtained from the impulse response through integration:

(5) $$\Theta \left(t\right)=\int_{0}^{t}\theta \left(t\right)\mathrm{dt}$$

**Figure 1:**
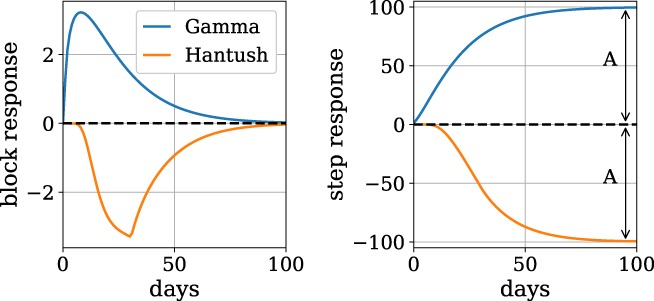
The one‐day block response and the step response functions for the scaled Gamma response function with parameters *A* = 100, *n* = 1.5, and *a* = 15 days and the Hantush response function with parameters *A* = 100, *b* = 4, and *a* = 15.

The step response corresponding to the scaled Gamma impulse response function (Equation 3) can be integrated analytically. The step response corresponding to the Hantush impulse response function is computed here using the approximation of Veling and Maas (2010). The step response eventually reaches a steady state value. For the Gamma function, the steady value due to a unit stress equals *A*, while for the Hantush function, the steady response equals‐*A* (a positive discharge results in lower heads). Examples of the block response and step response functions for the scaled Gamma and the Hantush functions are shown in Figure 1.

A stress can also be the combination of multiple stresses. An example of this is the linear model that is often used to simulate the net groundwater recharge *R*(*t*) from the rainfall *P*(*t*) and reference evaporation *E*_*r*_(*t*) (e.g., von Asmuth et al. 2008):

(6) $$R\left(t\right)=P\left(t\right)-{\mathrm{fE}}_{r}\left(t\right)$$

where *f* is a parameter. The net recharge *R*(*t*) is substituted for *S*_*m*_ in Equation 2 and convoluted with a response function to obtain the effect of recharge on the head. This approach to compute the net recharge works well for shallow groundwater levels in temperate climates with negligible runoff (e.g., Zaadnoordijk et al. 2018). For observation wells in more arid climates or in areas with deeper groundwater tables, recharge can not be estimated as a linear relationship of rainfall and reference evaporation, but a more complicated recharge model is needed (e.g., Berendrecht et al. 2004; Peterson and Western 2014).

The residuals of TFN models of heads often exhibit strong autocorrelation, violating the conditions that enable statistical inferences (e.g., parameter uncertainties and uncertainty of simulated heads). The residuals are modeled with a noise model (hence the name TFN modeling). An autoregressive model of order one is often used for time series with constant timesteps (Hipel and McLeod 1994). The equivalent for a time series with irregular timesteps is a noise model with exponential decay of the residuals (e.g., von Asmuth and Bierkens 2005):

(7) $$r\left(t_{i}\right)=\upsilon \left(t_{i}\right)+r\left(t_{i-1}\right)e^{-\Delta t_{i}/\alpha }$$

where *α* is the decay parameter, Δ*t*_*i*_ is the timestep between observations at *t*_*i*_ and *t*_*i* − 1_, and *υ*(*t*_*i*_) is the (approximate) white noise that is the result of a random process. The parameters of the TFN model may be estimated by optimizing an appropriate objective function, for example the sum of weighted squared noise (e.g., von Asmuth and Bierkens 2005). Many different methods are available to estimate optimal parameters and their uncertainty, varying from nonlinear least squares to Bayesian analysis.

## TFN Modeling with Pastas

In this section, it is described how TFN models are implemented in the Pastas software. The typewriter font (e.g., python_function) is used to refer to Python code. Information on how to download Pastas is given in the Acknowledgment section at the end of this paper.

### Object‐Oriented Design

**Figure 2:**
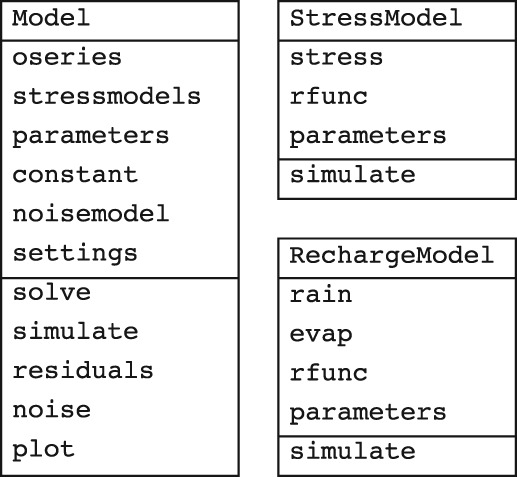
Unified modeling language (UML) diagrams of the three Pastas classes outlined in this section. Each diagram shows three boxes, from top to bottom: the class name, the main attributes, and the main methods.

The object‐oriented design of Pastas is relatively simple. Unified modeling language (UML) diagrams for the three most important classes are shown in Figure 2. The main class of the Pastas code is the Model class. A Model object stores the observed head series (oseries), keeps track of all the stresses and response functions that cause the head variations (stressmodels), the parameters in the model, the base level *d* (constant), the noise model, and basic model settings.

The model components to translate a stress (or two stresses in case of recharge) to a head contribution *h*_*m*_(*t*) are called stress models in Pastas. Each stress is stored separately in a StressModel object, together with the type of response function *θ*_*m*_ that is used to simulate the head response. Different response functions are available, including Exponential, Gamma, and Hantush. Each StressModel has a method to compute the contribution of that specific stress to the head variation, for example using convolution (Equation 2), or another method.

A specific stress model class is available for the effect of groundwater recharge. This class is called RechargeModel and simulates the combined effect of rainfall and reference evaporation by using Equation 4 to compute the net groundwater recharge. Nonlinear concepts for the calculation of groundwater recharge (e.g., Berendrecht et al. 2004; Peterson and Western 2014) are planned for a future version of Pastas. A RechargeModel object stores two stresses, the rainfall and (reference) evaporation time series, and one response function.

The five main methods of the Model class are (1) a method to estimate the parameters in the model, (2) a method to compute the simulated heads, (3) a method to compute the residuals, (4) a method to compute the noise, and (5) a basic method to plot the results. The method to compute the simulated heads is an implementation of Equation 1. The method loops through all *M* stress models and adds their contributions *h*_*m*_(*t*) to the head variations, then adds a base level *d*. The method to compute the residuals *r*(*t*) subtracts the simulated heads from the observed heads. The noise method applies Equation 7 to the residuals to compute the noise *υ*(*t*).

### The Pastas Package and Modeling Workflow

Pastas builds heavily on Pandas, a data analysis package for Python that efficiently deals with time series data (McKinney 2010). All time series need to be specified as Pandas Series or DataFrame objects. Observed heads may be measured at arbitrary times. Pastas uses regularly spaced stress series in the simulations, and has functionality to convert irregular time series to regularly spaced series.

The basic workflow of a Pastas model is as follows:

1. Import the Pastas package.
2. Read the time series from files and store them as Pandas Series or DataFrame objects.
3. Create a Model object and supply the observed head series.
4. Create StressModel objects by supplying the observed stress and specifying a response function, and add each StressModel object to the Model object.
5. Estimate the parameters of the Model and compute fit statistics.
6. Visualize output.
7. Analyze residuals and noise.

Each of the Steps 3 to 6 has a number of default settings that may be changed interactively, as demonstrated in the next sections. Pastas has separate subpackages for visualization and statistical analysis. The visualization capabilities include plotting of the model fit including contributions of the different stresses, and plotting of the response function(s). The statistical capabilities include computation of various measures of the model fit (e.g., *r* ^2^, root mean squared error [RMSE], and the Nash‐Sutcliffe coefficient), the Akaike and Bayesian information criteria, and a function to compute the autocorrelation of the residuals or the noise that deals with irregular time steps (based on Rehfeld et al. 2011). Other metrics to evaluate the model fit are available in Hydrostats (Roberts et al. 2018), a Python package that includes over 70 metrics to compare hydrological time series, working seamlessly with Pastas.

### Parameter Estimation

The default method in Pastas to estimate the parameters is to apply a nonlinear least squares algorithm to minimize the sum of weighted squared noise, according to von Asmuth and Bierkens (2005). It is important to choose reasonable initial values of the parameters to improve performance of the least squares algorithm. Initial values are estimated from the data when possible. For example, the initial value for the constant *d* in Equation 1 is estimated as the mean of the observed heads *h*(*t*), and the initial value for the scaling parameter of the response functions (e.g., *A* in Equations 3 and 4) are computed as 1/*σ*_*s*_, where *σ*_*s*_ is the standard deviation from the stress time series. Pastas includes methods to set different initial values for any of the parameters.

Parameter bounds are specified based on a physical interpretation of the parameters. For example, the scaling parameter *A* of the Gamma response functions has a lower bound of zero, so that the heads will go up in response to positive recharge. The evaporation factor *f* is bounded by 0 and −2, so that the actual evaporation can vary between zero and twice the reference evaporation. Pastas includes methods to set different minimum and maximum values for any of the parameters. In addition, it is possible to fix a parameter to its initial value. This can be helpful for parameters that do not significantly affect the outcome of the model.

Pastas currently includes only one option for the objective function to be minimized and two options to estimate the parameters while several others are under development. The present default is Scipy's least squares method, which uses a Trust Region Reflective algorithm to minimize the objective function and works well with bounds (Branch et al. 1999); this method is used in the first example. The other option is the LmFit solver (Newville et al. 2019), using Levenberg‐Marquardt minimization including bounds on the parameters; this method is used in the second example.

## Example 1: A Cookbook Recipe to Analyze Measured Heads Using Pastas

A Python script must be written for the seven steps to build a Pastas model defined in the previous section. In this first example application, it is shown how a simple TFN model is constructed using Pastas in a few lines of Python code. The objective is to investigate how well the heads measured in an observation well near Kingstown, Rhode Island, can be simulated using rainfall and reference evaporation.

The heads (site id 412918071321001) are obtained from the Groundwater Climate Response Network (CRN) of the USGS (downloaded from https://groundwaterwatch.usgs.gov). While the heads are available on a daily basis, only biweekly observations are used in this example for demonstration purposes. The rainfall data is taken from the Global Summary of the Day dataset (GSOD) available from the National Climatic Data Center (NCDC) for Kingston station (station number, NCDC WBAN: 54796), located at 41.491^°^, −71.541^°^. The reference evaporation is estimated using Thornthwaite's method (Pereira and Pruitt 2004) from the daily mean temperature for the same location.

The following steps describe the Python code used to create a Pastas model and analyze the heads:

1. **Import the Pastas package**. Import the Pandas and Pastas packages and, if desired, give them short aliases.

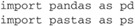

2 **Read the time series**. Time series can be imported using a variety of methods and need to be transformed into a Pandas Series. The easiest approach is probably to use the read_csv method of Pandas, which can be used to read almost any comma‐separated file.

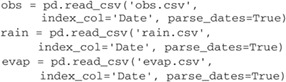


**Figure 3:**
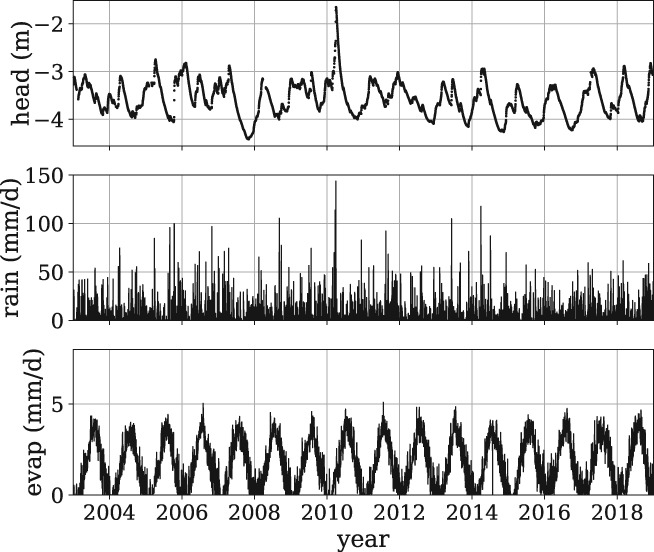
Time series of the observed heads, precipitation, and reference evaporation for Example 1.

The time series of the heads, rainfall and reference evaporation are stored in the variables obs, rain, and evap, respectively, and are plotted in Figure 3.

3 **Create a model object.** A Model instance is created and stored in the variable ml, where the observed heads obs and a name are the input arguments.

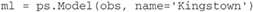


4 **Add stress models.** A RechargeModel instance is created and stored in the variable rm, taking the rainfall and reference evaporation time series as input arguments, as well as a name and a response function. In this example the Gamma response function is used (accessible as ps.Gamma). After creation, the RechargeModel instance is added to the model.

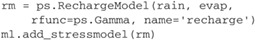

5 **Estimate model parameters.** The model parameters are estimated by calling the solve method of the Model instance:

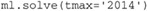


**Figure 4:**
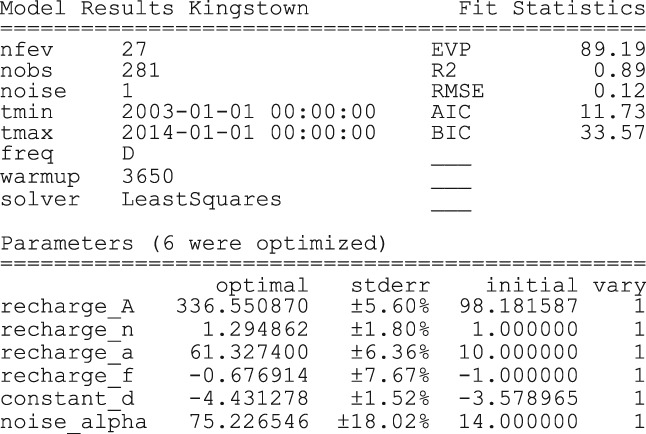
Fit report returned by the solve method, showing information on the model settings, the fit statistics and the estimated parameters.

In this example, tmax=‘2014’ is used, meaning that the model is calibrated on the available head observations up to 2014. A noise model and Scipy's least squares method (implemented as ps.LeastSquares) are used to estimate the parameters (default option). The solve method returns a report summarizing the model settings, the model fit, and the optimal parameter values and their estimated uncertainties, as shown in Figure 4 (default option).

6 **Visualize model results.** The results can be visualized with any of the predefined plotting methods, for example, the basic plot method. Here, the input argument tmax=‘2018’ is used to simulate and plot the heads up to 2018 (the default is to plot for the calibration period only).

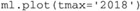


7 **Analyze residuals and noise.** The residuals, noise, the autocorrelation of the noise, and a few other subplots to analyze the residuals and noise may be plotted with the following command:

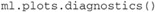


**Figure 5:**
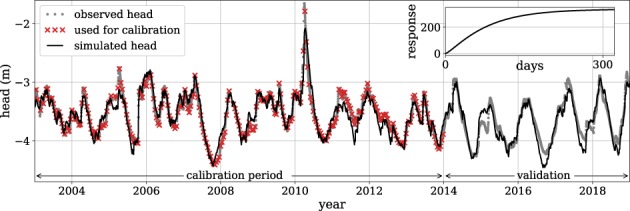
Plot of the simulated (black line) and observed heads (red crosses for those used during calibration, gray dots for unused heads), and the estimated step response (inset) for Example 1.

The plot resulting from Step 6 is shown in Figure 5 (with small modifications for this publication). The model simulation shows a good fit with the observed heads in the calibration period, supported by a low RMSE of 12 cm, and a high Pearson *r* ^2^ value of 0.89 (see fit report in Figure 4). The model also performs well in the validation period with a RMSE of 13 cm and *r* ^2^ of 0.84 for that period.

The estimated step response is shown in the inset of Figure 5 (added for this publication and not part of ml.plot()). The step response shows that the final level of 337 is reached after ∼300 d, which means that if it rains continuously 1 mm/d, the head in the observation well will eventually rise 337 mm after ∼300 d.

The observed head shows a peak in 2010 that is underestimated by the TFN model. The peak follows an extremely high rainfall event caused by the northeastern storms in New England in March of 2010. Apart from this extreme event, the observed heads can be simulated well with rainfall, reference evaporation (estimated from temperature data), and a simple TFN model.

**Figure 6:**
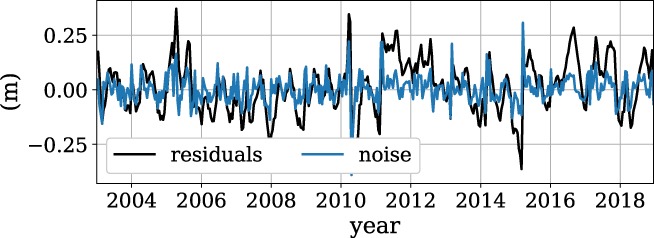
Plot of the residual and noise series of the model of Example 1.

**Figure 7:**
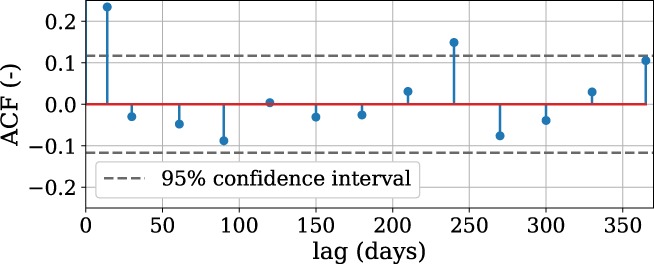
Autocorrelation plot for the noise of Example 1.

The residuals and noise are shown in Figure 6, and the autocorrelation plot of the noise is shown in Figure 7. Both plots are part of the diagnostics plot that is created in Step 7, but are shown separately for this publication. The residuals can indicate if a stress is missing from the model, for example when a clear trend in the residuals is visible. As expected, the residuals are autocorrelated with extended periods where the modeled heads are higher or lower than the observed heads, while the noise shows much less autocorrelation. Based on the autocorrelation graph (Figure 7) and the Ljung‐Box test (Ljung and Box 1978) it is concluded that there is no significant (*α* = 0.05) autocorrelation in the noise, so that statistical inferences can be made about the model output.

## Example 2: Estimate Drawdown Caused by Groundwater Pumping

In this second example, it is demonstrated how scripts can be used to analyze a large number of time series. Consider a pumping well field surrounded by a number of observations wells. The pumping wells are screened in the middle aquifer of a three‐aquifer system. The objective is to estimate the drawdown caused by the groundwater pumping in each observation well.

**Figure 8:**
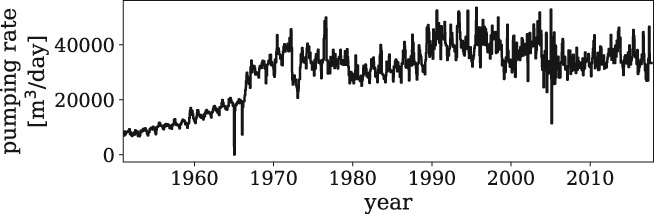
Time series of the pumping rate of the well field of Example 2.

**Figure 9:**
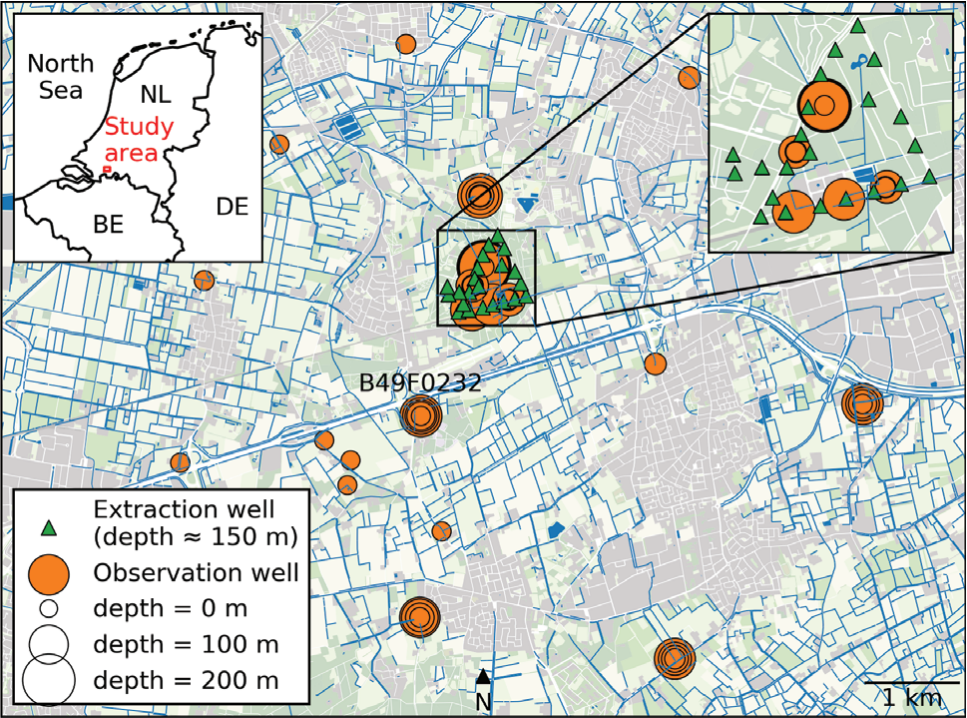
Map of the study area of Example 2. The green triangles are the 21 pumping wells. The orange circles are the 44 observations wells (some of them nested), where the circle size indicates the depth of the well screen below surface level.

Water extracted by the well field is used for drinking water supply in the southern part of the Netherlands. The well field consists of 21 pumping wells with screen depths between 110 and 170 m below surface level. A time series of the total extraction rate of all 21 pumping wells combined is available for modeling (Figure 8). The average total extraction rate calculated over the period 2007‐2018 is 34790 m^3^/d, or 12.7 · 10^6^m^3^/year. The surrounding monitoring network consists of 44 observation wells at 23 locations (some are nested wells with screens at different depths) (Figure 9). Daily rainfall measurements are obtained from weather station Oudenbosch, and Makkink reference evaporation (de Bruin and Lablans 1998) is obtained from weather station de Bilt, both operated by the Royal Dutch Meteorological Institute (KNMI, https://www.knmi.nl).

A Pastas model is created for each observation well using rainfall, reference evaporation, and pumping discharge as input series. Recharge is modeled with Equation 6. The response to recharge is modeled with the Gamma response function and the response to pumping with the Hantush response function.

**Figure 10:**
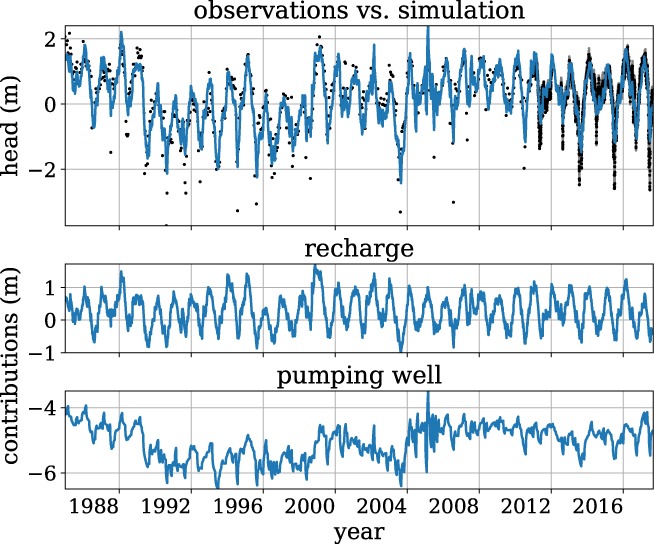
Time series model (blue line) of observed heads (black dots) at well B49F0232 (screen 5) for 1985‐2010 (top), with contributions of recharge (middle) and pumping (bottom).

As an example of one of the resulting models, consider well B49F0232 (Screen 5), located at ∼1500 m from the center of the well field. The model fit and the contributions of the net recharge and the pumping are shown in Figure 10. The model is fit for the entire period of record (1971‐2018), but only the period 1985‐2018 is shown. The pumping results in a lowering of the head between 4 and 7 m over the period shown. The net recharge contributes to the annual head variations and the pumping wells contribute mainly to the long‐term variations, most notably the lowering of the heads around 1990. The model underestimates the lows in the observed heads. Lower heads may be the result of nonlinear processes in the root zone that are not represented in the recharge model, or groundwater withdrawals for irrigation that are not included in the model.

Using a Python script, 44 Pastas models are created (one for each observation well) that include the effect of pumping. After the parameters are estimated, it is checked whether the estimated steady contribution of the pumping response (the scale parameter *A* in Equation 4) is significantly different from zero by checking whether the 95% confidence interval includes zero:

(8) $$|A|>1.96\sigma_{A}$$

where *σ*_*A*_ is the estimated standard deviation of parameter *A*. The contribution of pumping could not be determined with statistical significance for 9 of the 44 observation wells; these nine wells were not considered further.

**Figure 11:**
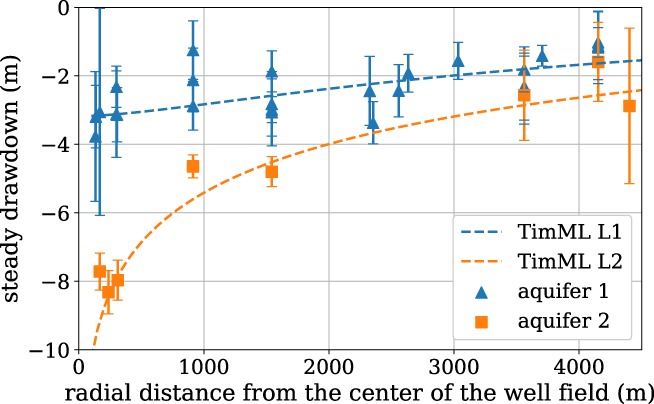
Estimated steady state drawdown for a pumping rate of 12.7 · 10^6^m^3^/year vs. the distance from the center of the well field of Example 2. The pumping wells are screened in Aquifer 2. The error bars denote the 95% confidence intervals of the drawdowns estimated with TFN models. The dashed lines are the result of the TimML model.

An advantage of many observation wells at different locations is that the results can be analyzed spatially and compared to the results of other (independent) estimates of the drawdown. The steady state drawdown for the average pumping rate over 2007‐2018 is computed for each observation well and plotted versus the distance of the observation well from the center of the well field in Figure 11. The markers in Figure 11 indicate in which aquifer each observation well is screened (three wells screened in the deep aquifer are not shown in the Figure). The drawdown is separately computed with the multilayer analytic element model TimML (version 6.0, Bakker and Strack 2003) using aquifer parameters within the range of values reported by the Dutch geological survey (Vernes et al. 2005), except for the transmissivity of the second aquifer for which a slightly higher transmissivity is used. The results of the TimML model are shown with the dashed lines in Figure 11 and are in good agreement with the drawdowns estimated with the TFN models.

## Conclusions and Discussion

Pastas is an open source Python package for the analysis of hydrogeological time series. The two objectives of Pastas are to provide a framework for developing and testing new modeling concepts and to provide ready‐to‐use software for practitioners. Pastas applies TFN modeling using predefined response functions. Multiple stresses (e.g., rainfall or pumping) and corresponding response functions (e.g., Gamma or Hantush) may be added to a Pastas model. Models are created and analyzed through scripts, which makes the modeling process transparent and provides a full record of the entire modeling process. A Pastas model can be created in seven steps. These seven steps were illustrated (including corresponding Python code) in the first example, where the heads from an observation well in Kingstown, Rhode Island are analyzed. The second example demonstrated how a large number of time series models can be built in batch; the results were analyzed spatially to determine the drawdown caused by a well field.

Pastas includes a large set of functionality, varying from a number of different response functions and the ability to select the calibration period to visualization and analysis of the results. All model concepts that have been implemented so far are for linear models: when the stress is doubled, so is the resulting head variation. This works very well for systems that behave more or less linearly, for example, relatively shallow (a few meters from the surface) wells in temperate climates. Pastas needs to be extended to be able to deal with other systems. For example, for deeper groundwater tables, the recharge is likely a nonlinear function of rainfall and evaporation (e.g., Berendrecht et al. 2004; Peterson and Western 2014). In arid regions, evaporation cannot be modeled as a (constant) fraction of reference evaporation (Equation 6). In more undulating terrain, rainfall may need to be adjusted for runoff. In dry periods, response functions may change when ditches and streams dry up (e.g., Knotters and Gooijer 1999). Many of these nonlinear modeling concepts will be explored for inclusion in Pastas in the coming years.

Many other topics deserve further investigation and development of which three are mentioned here. First, more advanced noise models are needed, because the simple first‐order noise model (Equation 7) does not always work well for high frequency data (daily or more frequent) and may even harm parameter estimation. Second, the current least squares objective function needs to be extended to include weighting of observations and alternative objective functions (e.g., maximum likelihood functions) need to be explored. Third, more accurate parameter estimation including estimation of the uncertainty of the parameters and model output is needed. Many alternative approaches are available for testing, including global searches and Bayesian approaches. The object‐oriented structure of Pastas is intended to facilitate the implementation and testing of many of these ideas and modeling concepts. The authors welcome new code contributions, use cases, and other suggestions from the community to improve and increase the use of TFN modeling in groundwater studies.

## Authors' Note

The author(s) does not have any conflicts of interest.
